# Leaching Thermodynamics of Low-Grade Copper Oxide Ore from [(NH_4_)_2_SO_4_]-NH_3_-H_2_O Solution

**DOI:** 10.3390/ma17194821

**Published:** 2024-09-30

**Authors:** Faxin Xiao, Xinyu Cao, Xuwei Luo, Ganfeng Tu, Cuixia Yang, Yu Peng, Hui Li, Wei Xu, Shuo Wang

**Affiliations:** 1 School of Metallurgy, Northeastern University, Shenyang 110819, China; 2271614@stu.neu.edu.cn (X.C.); 20203208@stu.neu.edu.cn (X.L.); 2271821@stu.neu.edu.cn (C.Y.); 1801555@stu.neu.edu.cn (Y.P.); 2 Key Laboratory for Recycling of Nonferrous Metal Resources (Shenyang), Shenyang 110819, China; 3 Key Laboratory for Ecological Metallurgy of Multimetallic Mineral (Ministry of Education), Northeastern University, Shenyang 110819, China; 4 China Nonferrous Metal Innovation Research Institute (Tianjin) Co., Ltd., No. 86 Ziguang Road, Zhongbei Industrial Park, Xiqing District, Tianjin 300380, China; huil65@163.com (H.L.); 13971787774@139.com (W.X.); wangs19981210@163.com (S.W.)

**Keywords:** ammonia leaching, ammonium sulfate, low-grade copper oxide, tenorite, leaching thermodynamic

## Abstract

This paper describes a highly alkaline low-grade copper oxide ore. Copper can be selectively leached out while other metals are retained. A thermodynamic model of the CuO-(NH_4_)_2_SO_4_-NH_3_-H_2_O system was established for the leaching of tenorite (CuO) under conditions of mass and charge conservation. MATLAB’s fitting functions, along with the diff and solve functions, were used to calculate the optimal ammonia concentration and total copper ion concentration of tenorite under different ammonium sulfate concentrations. The effects of various ammonia–ammonium salt solutions (ammonium sulfate, ammonium carbonate, ammonium chloride) on the copper leaching rate were investigated. Results show that under the conditions of an ammonia concentration of 1.2 mol/L, an ammonia–ammonium ratio of 2:1, a liquid–solid ratio of 3:1, a temperature of 25 °C, and a leaching time of 4 h, the copper leaching rate from the ammonium sulfate and ammonium chloride solutions reaches 70%, which is slightly higher than that of ammonium carbonate. Therefore, an ammonia–ammonium sulfate system is selected for leaching low-grade copper oxide due to its lower corrosion to equipment compared to the chlorination system. The impact of this study on industrial applications includes the potential to find more sustainable and cost-effective methods for resource recovery. The industry can reduce its dependence on resources and mitigate its environmental impact. Readers engaged in low-grade oxidized copper research will benefit from this study.

## 1. Introduction

Copper is an important strategic metal resource widely used in electronic, electrical, and mechanical manufacturing and other fields because of its advantages in mechanical properties, conductivity, and heat conduction [1,2]. For years, copper has been the second non-ferrous metal after aluminum. Due to its huge demand and China’s severe shortage of copper resources, China needs to import a large amount of copper concentrate and refined products from abroad every year [3,4]. The main difference between high-grade copper oxide and low-grade copper oxide lies in their copper content and selectivity. High-grade copper oxide has a higher proportion of copper and is more economically valuable for mining operations. With the continuous consumption of high-grade copper sulfide around the world, it is imperative to utilize refractory low-grade copper oxide [5,6].

High-alkaline gangue type low-grade copper oxide deposits are typical examples of low-grade copper oxide deposits with relatively abundant reserves, primarily found in the Democratic Republic of Congo (DRC) and Zambia [7]. In China, these deposits are mainly distributed in Xinjiang, Yunnan, Sichuan, Inner Mongolia, etc., with notable examples such as the Dongchuan oxidized copper deposit in Yunnan, Leshan oxidized copper deposit in Sichuan, and sandstone oxidized copper deposit in Xinjiang [8]. These minerals are characterized by “three high and one low”: high alkaline gangue content, high oxidation rate, high mud content, and low grade, representing typical refractory low-grade oxidized copper ore [9,10]. Specific characteristics include: (1) impurities such as calcium, magnesium, and aluminum in the ore, with the alkaline gangue formed by calcium and magnesium accounting for around 20%~40% of the ore, contributing to its high alkalinity; (2) predominantly oxide minerals in the ore, with high oxidation and binding rates; (3) high SiO_2_ content, approximately 20%; (4) low copper grade, with Cu grade less than 1%. These ores may contain high levels of impurities and complex mineralogy, making separation and leaching challenging. Additionally, they may possess high hardness or abrasiveness, complicating effective grinding and leaching.

For high-alkaline gangue type low-grade copper oxide, using the common sulfuric acid leaching process, typically applied to other copper oxide ores [8], results in high acid consumption. This is because when metal sulfide ores are leached with sulfuric acid, metal sulfides oxidize to form metal sulfate and sulfur dioxide, consuming a significant amount of acid and causing equipment corrosion. This results in minimal economic benefits, and large quantities of calcium sulfate generated during leaching are adsorbed on the ore heap, leading to bonding phenomena that severely degrade the subsequent solution penetration effect. By using the ammonia leaching process, it is possible to avoid the dissolution of high-alkaline substances like calcium and magnesium into the solution, ensuring the purity of copper in cuprammonium solution [11,12] and achieving leaching efficiency comparable to acid leaching. Currently, most of these minerals are immersed in ammonia [13,14], but the ammonia concentration in these studies is high, exceeding 2 mol/L. The high concentration of ammonia causes electrochemical corrosion with reaction equipment and is prone to volatilization, which can deteriorate the operating environment, making large-scale industry application challenging [15]. To prevent serious corrosion, researchers select corrosion-resistant metals such as stainless steel and nickel alloys, which have strong resistance but also increase leaching costs. Therefore, studying the mechanism and process of ammonia leaching for industrial applications of low-grade copper oxide with high-alkaline gangue is of great significance.

In this paper, refractory high-alkaline gangue low-grade copper oxide ore is the research object, focusing on the copper-bearing mineral tenorite (CuO). A thermodynamic model of the CuO-(NH_4_)_2_SO_4_-NH_3_-H_2_O system was established in the leaching system based on mass and charge balance equations, and thermodynamic calculations were performed. On this basis, different ammonia–ammonium leaching systems were verified through experiments. This study achieved efficient copper ion leaching under relatively low ammonia concentration conditions. Meanwhile, the anticipated challenges in scaling up the ammonia leaching process for industrial applications, considering ore characteristics and leaching behavior, require careful consideration and strategic planning. These challenges include water management, process control, stability, and the need for investment in specialized equipment and infrastructure for large-scale application.

## 2. Materials and Methods

### 2.1. Experimental Material

**Figure 1:**
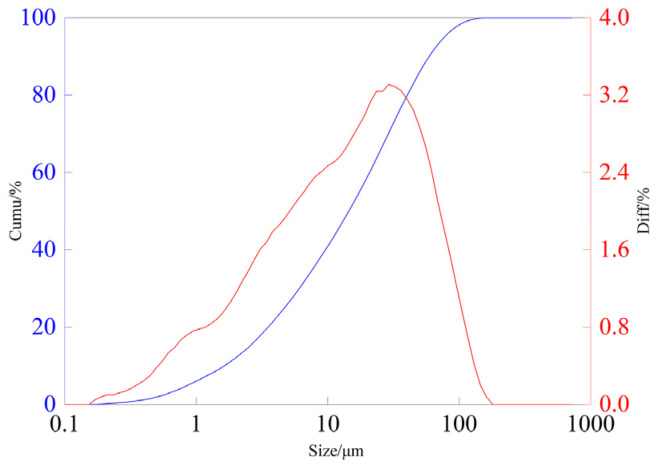
Grain size analysis results of ore samples. (red line represents differential distribution, blue line represents cumulative distribution).

The ore sample was taken from a high-alkaline gangue oxide copper mine in Congo Gold, and the main metallic minerals in the sample include tenorite (CuO), malachite (Cu_2_(CO_3_)(OH)_2_), etc. Gangue ore mainly consists of quartz and calcite, and the X-ray fluorescence (ZSXPrimus Ⅱ, Saitama, Japan) results of the ore samples are listed in Table 1. As shown in Table 1, the grade of copper in the raw ore is low, and the gangue is mainly composed of silica, calcium oxide, magnesium oxide, and aluminum oxide, which is a typical low-grade copper oxide with high-alkaline gangue. By ICP (Thermo Fisher Scientific, Waltham, MA, USA) quantitative analysis, the Cu content of ore samples is only 0.89%. Particle size analysis was carried out on the ore sample, and the results are shown in Figure 1.

As shown in Figure 1, the ore sample size distribution is as follows: particles smaller than 7.29 μm account for 33.85%, and particles between 7.29 and 74.12 μm account for a significant proportion of 60.52%.

### 2.2. Experimental Method

Because the ore sample contains a lot of calcium salt and magnesium salt, the leaching channel is blocked. If acid leaching is used, it will prolong the leaching cycle, reduce the leaching efficiency, and increase the production cost. If acid leaching (dilute sulfuric acid) is used directly, micro-soluble calcium sulfate and magnesium sulfate will be produced in the leaching process and block the leaching channel. Therefore, ammonia leaching was used in the experiment. The procedure is as follows:

Weigh 20 g of mineral powder into a 250 mL conical flask, add a certain concentration of ammonia–ammonium sulfate solution, and heat it in a water bath with a heat collecting magnetic stirrer. After the temperature reaches the set point, keep it constant, and start stirring and timing. After the reaction, the leaching solution was pumped and filtered to 50 mL. Through experimental design, the mass concentration of copper was analyzed using a spectrophotometer, and the copper leaching rate was calculated. During the experiment, the thermometer was fixed on one side of the conical bottle to monitor the temperature during the experiment. The accuracy of temperature control was 0.1 °C.

## 3. Results

### 3.1. Influence of Ammonia Concentration on Copper Leaching Rate 

**Figure 2:**
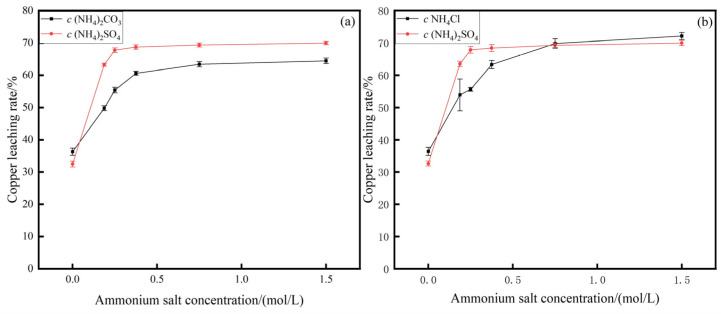
Comparison of influence of different systems on copper leaching rate. System: (**a**) ammonium carbonate and ammonium sulfate; (**b**) ammonium chloride and ammonium sulfate.

Reaction conditions: ammonia concentration 1.2 mol/L, stirring intensity 250 r/min, liquid–solid mass ratio 2:1, temperature 25 °C, leaching time 4 h, copper leaching rate with different ammonium salt concentrations in the relationship is shown in Figure 2. The optimal copper leaching rate reached 70%.

At present, the price of ammonium sulfate and ammonium carbonate is Chinese Yuan (CNY) 900 per ton, slightly higher than the CNY 570 per ton of ammonium chloride. This is because ammonium sulfate contains sulfur element and can be used as sulfur fertilizer, which increases its value. Ammonium carbonate is a fertilizer that can quickly releases nitrogen. In contrast, the production cost of ammonium chloride is lower and the production process is simple.

As shown in Figure 2, the copper leaching rate increases rapidly with the concentration of ammonium carbonate and ammonium chloride and then gradually becomes stable. When the concentration of ammonium carbonate is 0.8 mol/L, the copper leaching rate can reach 63.5%. When the concentration of ammonium chloride is 0.8 mol/L, the copper leaching rate can reach 70.0%. Under other conditions, when the concentration of ammonium sulfate is 0.8 mol/L, the copper leaching rate can reach 68.9%. Compared with other studies, the concentration of ammonium sulfate used in this study is even lower [16]. The Cu leaching rate using ammonium carbonate is lower than that of ammonium sulfate and ammonium chloride. In the beginning, the leaching rate using ammonium chloride is lower than that of ammonium sulfate, and reaches nearly the same value under high concentration of ammonium salt. While However, ammonium chloride is highly corrosive to the equipment, so the ammonia–ammonium sulfate system is chosen. Ammonium chloride and ammonium sulfate can enhance the solubility of certain metals in the leaching solution through specific chemical interactions, such as the formation of soluble metal–ammonium complexes. In contrast, CO_3_^2−^ ions bind with metal ions, leading to a decrease in leaching efficiency.

### 3.2. Structure Characterization of Leaching Residue

**Figure 3:**
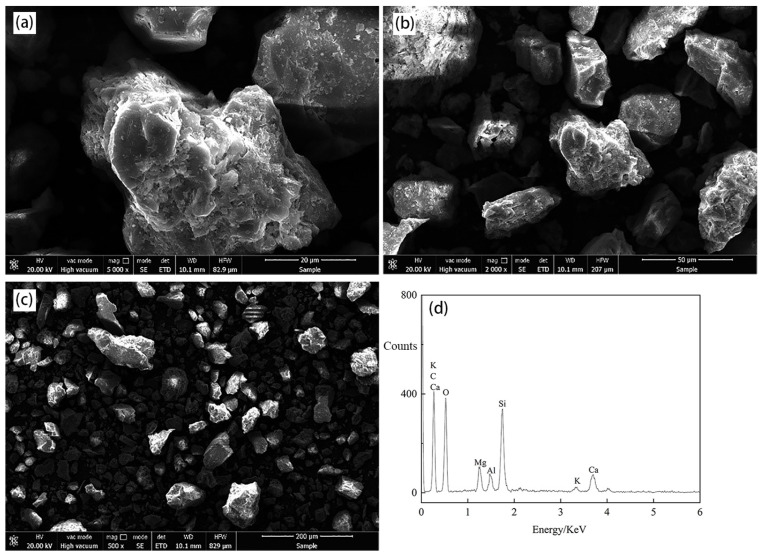
Surface morphology of the ore samples after leaching at different proportions. (**a**) 20 μm; (**b**) 50 μm; (**c**) 200 μm; (**d**) energy spectrum.

The raw copper oxide ore was leach for 4 h under the conditions of ammonia concentration of 1.2 mol/L, ammonium sulfate concentration of 0.6 mol/L, liquid–solid mass ratio of 2:1, stirring intensity of 250 r/min, and temperature of 25 °C. SEM-EDS analysis (ULTRA-PLUS, Zeiss, Oberkochen, Germany) was conducted on the leaching residue after the end of leaching, and the surface morphology of raw ore before leaching was compared. The result is shown in Figure 3.

As shown in the surface topography of the ore sample in Figure 3, the ore sample was eroded by leaching agents and its surface was rough and uneven, but the size and shape of the ore sample particles basically did not change. In Figure 3d, the content of Si, O, Ca, and Al elements is high, and according to ICP quantitative analysis, the mass fraction of Cu in the leaching slag measured is only 0.27%, indicating that a lot of the Cu element is leached in the ammonia–ammonium sulfate system, and a small amount of Cu element remains in the leaching slag. 

## 4. Discussion

### 4.1. Leaching Principle

The ammonia leaching method is the most effective leaching method for high-alkaline gangue and low-grade copper oxide [17]. The copper in the ore is dissolved into the solution by forming stable complex ions with ammonia, while impurities such as calcium and magnesium do not participate in the reaction, so that the target metal and impurities such as calcium and magnesium metal are separated. The advantages of the ammonia leaching method include a smaller amount of leaching agent needed, high selectivity, and high solution purity.

The main copper oxide ores are malachite and black copper, and the ammonia leaching equations are shown as follows [16].

(1) $${\mathrm{CuCO}}_{3}\cdot {\mathrm{Cu}\left(\mathrm{OH}\right)}_{2}+6{\mathrm{NH}}_{3}+2{\mathrm{NH}}_{4}^{+}=2{\mathrm{Cu}({\mathrm{NH}}_{3})}_{4}^{2+}+{2H}_{2}O+CO_{3}^{2-}$$

(2) $$2{\mathrm{CuCO}}_{3}\cdot {\mathrm{Cu}\left(\mathrm{OH}\right)}_{2}+10{\mathrm{NH}}_{3}+2{\mathrm{NH}}_{4}^{+}=3{\mathrm{Cu}({\mathrm{NH}}_{3})}_{4}^{2+}+2H_{2}O+2CO_{3}^{2-}$$

(3) $$\mathrm{CuO}+2{\mathrm{NH}}_{3}+{\;2\mathrm{NH}}_{4}^{+}={\;\mathrm{Cu}({\mathrm{NH}}_{3})}_{4}^{2+}+H_{2}O$$

### 4.2. Thermodynamic Analysis

In the low-grade copper oxide ore with highly alkaline gangue, the main copper phase is black copper. The thermodynamic model of CuO-(NH_4_)_2_SO_4_-NH_3_-H_2_O was established in the leaching system of [(NH_4_)_2_SO_4_]-NH_3_-H_2_O based on both mass balance and charge balance equations [18,19]. Since it is difficult to obtain the activity coefficient of each ion in the solution, molar concentration is used to replace the activity in the calculation in this paper. The stability constants of related complex ions in the system are shown in Table 2 [20].

As shown in Table 2, Cu^2+^ can form relatively complex coordination compounds with NH_3_ and OH^−^ ions in the solution. Among these, the stability of the complexes varies, with Cu(NH_3_)^2+^ and Cu(NH_3_)_2_^2+^ being the most stable forms.

#### Establishment of Thermodynamic Model

The equilibrium between copper-containing species and simple ions in the solution can be expressed by Equation (4).


(4) $${\mathrm{Cu}}_{i}{\left({\mathrm{NH}}_{3}\right)}_{j}{\left(\mathrm{OH}\right)}_{k}{\left({\mathrm{SO}}_{4}\right)}_{p}^{2i-k-2p}={\mathrm{iCu}}^{2+}+{\mathrm{jNH}}_{3}+{\mathrm{kOH}}^{-}+{\mathrm{pSO}}_{4}^{2-}$$

In the formula, i, j, k, and p are respectively the numbers of Cu^2+^, NH_3_, OH^−^, and SO_4_^2−^ in the coordinated ions, and their values are: i =1, 2; j = 0, 1, 2, 3, 4, 5; k = 0, 1, 2, 3, 4; p = 0, 1.

The stability constant expression of the complex is shown in Equation (5) [19].


(5) $$\frac{\left[{\mathrm{Cu}}_{i}{\left({\mathrm{NH}}_{3}\right)}_{j}{\left(\mathrm{OH}\right)}_{k}{\left({\mathrm{SO}}_{4}\right)}_{p}^{2i-k-2p}\right]}{{\left[{\mathrm{Cu}}^{2+}\right]}^{i}{\left[{\mathrm{NH}}_{3}\right]}^{j}{\left[{\mathrm{OH}}^{-}\right]}^{k}{\left[{\mathrm{SO}}_{4}^{2-}\right]}^{p}}={\;\beta }_{i,j,k,p}$$

The relationship between the available coordinated ions and their constituent simple ions is shown in Equation (6).


(6) $$\left[{\mathrm{Cu}}_{i}{\left({\mathrm{NH}}_{3}\right)}_{j}{\left(\mathrm{OH}\right)}_{k}{\left({\mathrm{SO}}_{4}\right)}_{p}^{2i-k-2p}\right]=\beta_{i,j,k,p}{\left[{\mathrm{Cu}}^{2+}\right]}^{i}{\left[{\mathrm{NH}}_{3}\right]}^{j}{\left[{\mathrm{OH}}^{-}\right]}^{k}{\left[{\mathrm{SO}}_{4}^{2-}\right]}^{p}$$

Three solid phases (CuO, Cu(OH)_2_ and Cu(OH)_1.5_(SO_4_)_0.25_) may exist in the CuO-NH_3_-(NH_4_)_2_SO_4_ system, because CuO and Cu(OH)_2_ have the same solubility product form. The existence of two stable solid phases can be determined by comparing the solubility product (Ksp) of CuO and Cu(OH)_2_. When CuO is assumed as the stable solid phase, the dissolution reaction is shown in Equation (7). When Cu(OH)_2_ is assumed as the stable solid phase, the solution equation is as shown in Equation (8).


(7) $$\mathrm{CuO}+{\;H}_{2}O={\;\mathrm{Cu}}^{2+}+{\;2\mathrm{OH}}^{-},K_{\mathrm{sp}}={\;10}^{-19.51}$$

(8) $${Cu\left(\mathrm{OH}\right)}_{2}={\mathrm{Cu}}^{2+}+{2\mathrm{OH}}^{-},K_{\mathrm{sp}}=10^{-18.9}$$

By comparing Equations (7) and (8), it can be seen that the Ksp of CuO is smaller than that of Cu(OH)_2_, indicating that CuO is more stable than Cu(OH)_2_ [21]. Even if solid Cu(OH)_2_ is formed during the dissolution process, it will be converted to CuO until the Cu(OH)_2_ disappears. This shows that there is no Cu(OH)_2_ solid phase in CuO-NH_3_-(NH4)_2_SO_4_ system with CuO [22,23,24].

According to Equation (7), Equation (9) is obtained.


(9) $$\left[{\mathrm{Cu}}^{2+}\right]=10^{-19.51}\cdot {\left[{\mathrm{OH}}^{-}\right]}^{-2}$$

By substituting Equation (9) into Equation (6), Equation (10) can be obtained.

(10) $$\begin{array}{c} \left[{\mathrm{Cu}}_{i}{\left({\mathrm{NH}}_{3}\right)}_{j}{\left(\mathrm{OH}\right)}_{k}{\left({\mathrm{SO}}_{4}\right)}_{p}^{2i-k-2p}\right]=\beta_{i,j,k,p}10^{-19.51i}\cdot {\left[{\mathrm{OH}}^{-}\right]}^{-2i}{\left[{\mathrm{NH}}_{3}\right]}^{j}{\left[{\mathrm{OH}}^{-}\right]}^{k}{\left[{\mathrm{SO}}_{4}^{2-}\right]}^{p}\\ =\exp\left(\ln\left(\beta_{i,j,k,p}10^{-19.51i}\right)+\mathrm{jln}\left[NH_{3}\right]+\left(k-2i\right)\ln10\cdot \left(\mathrm{pH}\;-\;{\mathrm{pK}}_{w}\right)+\mathrm{pln}\left[SO_{4}^{2-}\right]\right)\\ =\exp\left(\ln\left(\beta_{i,j,k,p}10^{-19.51i}\;-\;\left(k\;-\;2i\right)\ln10\cdot pK_{w}\right)+\mathrm{jln}\left[NH_{3}\right]+\left(k\;-\;2i\right)\ln10\cdot \mathrm{pH}+\mathrm{pln}\left[SO_{4}^{2-}\right]\right)\\ =\exp\left(A+\mathrm{Bln}\left[NH_{3}\right]+\mathrm{CpH}+\mathrm{Dln}\left[SO_{4}^{2-}\right]\right) \end{array}$$

where A = ln(β_i,j,k,p_10^ −19.51i^) − (k − 2i)ln10 ⋅ pK_w_; B = j; C = (k − 2i)ln10; D = p.

When the stable solid is Cu(OH)_1.5_(SO_4_)_0.25_, the dissolution reaction is as shown in Equation (11).


(11) $${\mathrm{Cu}\left(\mathrm{OH}\right)}_{1.5}{\left({\mathrm{SO}}_{4}\right)}_{0.25}={\;\mathrm{Cu}}^{2+}+{1.5\mathrm{OH}}^{-}+0.25{\mathrm{SO}}_{4}^{2-},{\;K}_{\mathrm{sp}}={\;10}^{-16.86}$$

According to Equation (11), Equation (12) is obtained.


(12) $$\left[Cu^{2+}\right]=10^{-16.86}{\left[OH^{-}\right]}^{-1.5}\cdot {\left[SO_{4}^{2-}\right]}^{-0.25}$$

Substitute Equation (12) into Equation (6) to obtain Equation (13).

(13) $$\begin{array}{c} \left[Cu_{i}{\left(NH_{3}\right)}_{j}{\left(\mathrm{OH}\right)}_{k}{\left(SO_{4}\right)}_{p}^{2i-k-2p}\right]=\beta_{i,j,k,p}10^{-16.86i}\cdot {\left[NH_{3}\right]}^{j}{\left[OH^{-}\right]}^{k-1.5i}{\left[SO_{4}^{2-}\right]}^{p-0.25i}\\ =\exp\left(\ln\left(\beta_{i,j,k,p}10^{-16.86i}\right)+j\ln\left[NH_{3}\right]+\left(k-1.5i\right)\ln10\cdot \left(\mathrm{pH}-pK_{w}\right)+\left(p-0.25i\right)\ln\left[SO_{4}^{2-}\right]\right)\\ =\exp\left(A+B\ln\left[NH_{3}\right]+\mathrm{CpH}+D\ln\left[SO_{4}^{2-}\right]\right) \end{array}$$

where A = ln(β_i,j,k,p_10^− 16.86i^) − (k − 1.5i)ln10 ⋅ pK_w_; B = j; C = (k − 1.5i)ln10; D = p − 0.25i.

The equilibrium equations of total copper, total ammonia, and charge in solution can be expressed as Equations (14), (15), and (16), respectively.

(14) $$\begin{array}{c} c{\left({\mathrm{Cu}}^{2+}\right)}_{T}=\left[{\mathrm{Cu}}^{2+}\right]+\sum_{i=1}^{5}\left[{\mathrm{Cu}\left({\mathrm{NH}}_{3}\right)}_{i}^{2+}\right]+\sum_{j=1}^{4}\left[{\mathrm{Cu}\left(\mathrm{OH}\right)}_{j}^{2-j}\right]+2\left[{\mathrm{Cu}}_{2}{\left(\mathrm{OH}\right)}_{2}^{2+}\right]\\ +\left[\mathrm{Cu}{\left({\mathrm{NH}}_{3}\right)}_{3}{\left(\mathrm{OH}\right)}^{+}\right]+\left[\mathrm{Cu}{\left({\mathrm{NH}}_{3}\right)}_{2}{\left(\mathrm{OH}\right)}_{2}\right]+\left[\mathrm{Cu}{\left({\mathrm{NH}}_{3}\right)}_{2}{\left(\mathrm{OH}\right)}_{3}^{-}\right] \end{array}$$

(15) $$\begin{array}{c} c{\left({\mathrm{NH}}_{3}\right)}_{T}=c\left({\mathrm{NH}}_{3}\right)+2\left[{\left({\mathrm{NH}}_{4}\right)}_{2}{\mathrm{SO}}_{4}\right]=\left[{\mathrm{NH}}_{4}^{+}\right]+\left[{\mathrm{NH}}_{3}\right]+\sum_{i=1}^{5}i\cdot \left[{\mathrm{Cu}\left({\mathrm{NH}}_{3}\right)}_{i}^{2+}\right]\\ +3\left[\mathrm{Cu}{\left({\mathrm{NH}}_{3}\right)}_{3}{\left(\mathrm{OH}\right)}^{+}\right]+2\left[\mathrm{Cu}{\left({\mathrm{NH}}_{3}\right)}_{2}{\left(\mathrm{OH}\right)}_{2}\right]+\left[\mathrm{Cu}{\left({\mathrm{NH}}_{3}\right)}_{2}{\left(\mathrm{OH}\right)}_{3}^{-}\right] \end{array}$$

(16) $$\sum m\cdot \left[X\right]=0$$

where c(Cu^2+^)T and c(NH_3_)T respectively represent the total concentration of copper and ammonia in the solution. The total concentration of ammonia in the solution is the sum of the concentration of ammonia added to the solution c(NH_3_) and twice the concentration of ammonium sulfate c[(NH_4_)_2_SO_4_]. “m” and “[X]” respectively represent the charge number and concentration of each ion in the solution. 

When the stable solid phase is CuO, the mass balance equation of total sulfate root can be expressed as Equation (17).

(17) $$c{\left({\mathrm{SO}}_{4}^{2-}\right)}_{T}=c\left[{\left({\mathrm{NH}}_{4}\right)}_{2}{\mathrm{SO}}_{4}\right]=\left[{\mathrm{SO}}_{4}^{2-}\right]$$

where c(SO_4_^2−^)T is equal to the concentration of total ammonium sulfate added to the solution.

When the stable solid phase is Cu(OH)_1.5_(SO_4_)_0.25_, the total sulfuric acid concentration in the solution comes from two parts [16]: per mole of ammonium sulfate added to the solution and per mole of Cu(OH)_1.5_(SO_4_)_0.25_ dissolved in the solution contribute 1 mol and 0.25 mol of sulfate ions, respectively, so the mass balance equation of total sulfuric acid concentration can be expressed as Equation (18).


(18) $$c{\left(SO_{4}^{2-}\right)}_{T}=c\left[{\left(NH_{4}\right)}_{2}SO_{4}\right]+0.25c\left(Cu^{2+}\right)=\left[SO_{4}^{2-}\right]$$

The expression [X] = exp(A + Bln[NH_3_] + CpH + Dln[SO_4_^2−^]) is split respectively into Equations (14), (15), (16), and (17) or (14), (15), (16), (18). It can be seen that there are six unknowns in the system of equations: c(Cu^2+^)T, c(NH_3_), c[(NH_4_)_2_SO_4_)], [NH_3_], pH, and [SO_4_^2−^]. During the experiment, the concentration of ammonia and ammonium sulfate is taken as the control condition to change between 0 mol/L and 5 mol/L. Only four variables, [NH_3_], pH, [SO_4_^2−^], and c(Cu^2+^), remain in the system of unknowns. It can be solved by calling the function “fsolve” in MATLAB software(R2019a) to obtain the stereogram of the changes in [NH_3_], pH, [SO_4_^2−^], and c(Cu^2+^) with c(NH_3_) and c[(NH_4_)_2_SO_4_)]. 

**Figure 4:**
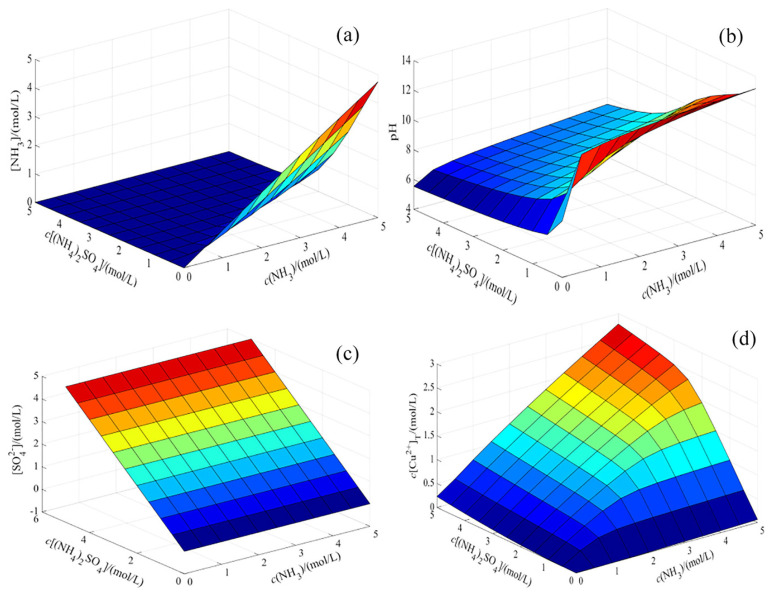
Equilibrium surface of different variables. Variables: (**a**) [NH3]; (**b**) pH; (**c**) [SO_4_^2−^]; (**d**) [c(Cu^2+^)].

The solution results of the thermodynamic model of the CuO-NH_3_-(NH_4_)_2_SO_4_-H_2_O system regarding [NH_3_], pH, [SO_4_^2−^], and [c(Cu^2+^)] are shown in Figure 4. 

As shown in Figure 4a–c, [NH_3_] is jointly controlled by c(NH_3_) and c[(NH_4_)_2_SO_4_], but it is primarily influenced by c(NH_3_), with c[(NH_4_)_2_SO_4_] playing a minimal role. The concentration of c(NH_3_) promotes an increase in pH, whereas the concentration of c[(NH_4_)_2_SO_4_] inhibits this increase. When c(NH_3_) ranges between 0 and 1 mol/L, there is a linear relationship between pH and c(NH_3_). Free sulfate ions are provided by c[(NH_4_)_2_SO_4_] and their concentration increases with the rise in c[(NH_4_)_2_SO_4_]. The surface relationships among total c(Cu^2+^)T, c[NH_3_], and c[(NH_4_)_2_SO_4_^2−^] are depicted in Figure 4d. While the variation relationship between c(Cu^2+^)T, c[NH_3_], and c[(NH_4_)_2_SO_4_^2−^] can be observed, it does not accurately reflect the magnitude of numerical changes.

(1) **Effects of c(NH_3_) and c[(NH_4_)_2_SO_4_] on the concentration of Cu^2+^**

**Figure 5:**
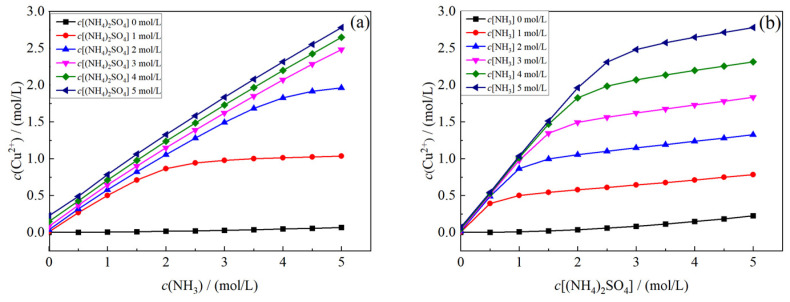
Change curve of total copper concentration under different variables. Variables: (**a**) c(NH_3_); (**b**) c[(NH_4_)_2_SO_4_].

The surface relations between c(Cu^2+^)T and c[NH_3_] and between c(Cu^2+^)T and c[(NH_4_)_2_SO_4_^2−^] are projected onto the c(NH_3_)–c(Cu^2+^)T plane and c[(NH_4_)_2_SO_4_]–c(Cu^2+^)T plane, respectively, as shown in Figure 5.

As shown in Figure 5a, the concentration of ammonium sulfate has a great influence on c(Cu^2+^)T. Increasing the concentration of ammonium sulfate is conducive to the improvement of c(Cu^2+^)T. When c[(NH_4_)_2_SO_4_] ≥ 3 mol/L, c(Cu^2+^)T increases linearly with the increase in c(NH_3_). According to Figure 5b, when the solution is pure ammonium sulfate solution, the solubility of copper oxide (CuO) is very small, that is, pure ammonium sulfate can hardly leach copper oxide (CuO), and c[(NH_4_)_2_SO_4_] only has a great influence on c(Cu^2+^)T when it is between 0 and 3 mol/L. After 3 mol/L, the further increase in c[(NH_4_)_2_SO_4_] decreases the increase in c(Cu^2+^)T gradually.

(2) **Stable region map**

**Figure 6:**
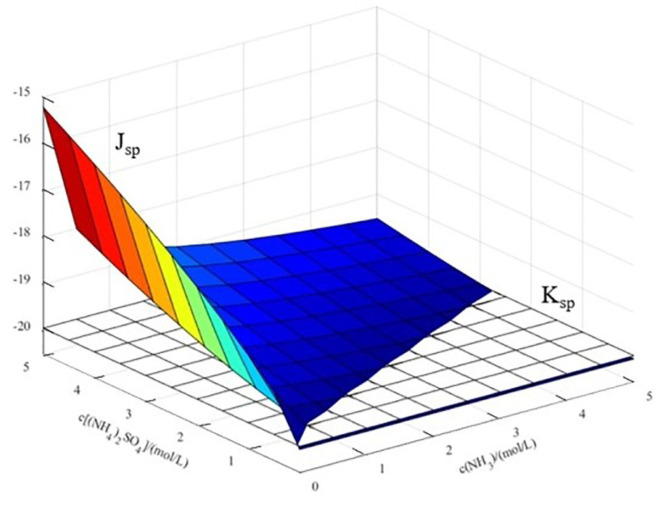
Comparison of J_sp_ and K_sp_ of Cu(OH)_1.5_(SO_4_)_0.25_.

In this system, there may be a case where the equilibrium solid phase is Cu(OH)_1.5_(SO_4_)_0.25_. As shown in Figure 6, it is easy to compare the size of J_sp_ and K_sp_ for Cu(OH)_1.5_(SO_4_)_0.25_.

As shown in Figure 6, when the ammonium sulfate concentration is high and the ammonia concentration is low, the J_sp_ of Cu(OH)_1.5_(SO_4_)_0.25_ is larger than K_sp_. In other words, CuO is transformed into Cu(OH)_1.5_(SO_4_)_0.25_, and this region represents the stable region of Cu(OH)_1.5_(SO_4_)_0.25_. Other regions are the stable regions of CuO. The thermodynamic model analysis results above are consistent with the thermodynamic study findings of Chen et al. [25], providing strong credibility.

## 5. Conclusions

Thermodynamic calculations for copper oxide in the ammonium–ammonium sulfate system indicate that [NH_3_] is controlled by both c(NH_3_) and c[(NH_4_)_2_SO_4_] during the ammonia leaching process of black copper ore, with c(NH_3_) being the primary contributor. c(NH_3_) promotes pH increase, while c[(NH_4_)_2_SO_4_] inhibits it. When c(NH_3_) ranges from 0 to 1 mol/L, pH varies linearly with c(NH_3_). Free sulfate ions are provided by c[(NH_4_)_2_SO_4_], increasing as its concentration rises. Ammonium sulfate regulates ammonia concentration and pH in this system [25,26].

Comparing the solubility products K_sp_ of CuO and Cu(OH)_2_ shows that CuO is the stable solid phase. However, at high ammonium sulfate concentrations and low ammonia concentrations, the species of Cu(OH)_1.5_(SO_4_)_0.25_ may be a stable solid phase.

Leaching experiments with different ammoniacal salts (ammonium sulfate, ammonium carbonate, and ammonium chloride) revealed that copper leaching rates increase rapidly with rising concentrations of ammonium carbonate and ammonium chloride before stabilizing. At 0.8 mol/L concentration:

Ammonium carbonate achieves a copper leaching rate of 63.5%.

Ammonium chloride achieves a rate of 70.0%.

Ammonium sulfate achieves a rate of 68.9%.

Ammonium chloride and ammonium sulfate have similar effects on leaching efficiency, both outperforming ammonium carbonate; however, due to its corrosiveness to equipment, ammonium chloride is less favorable than the ammonia–ammonium sulfate system.

The choice of leaching agent is very important to the leaching efficiency. In future studies, the leaching agent can be selected and optimized through experiments to improve the leaching efficiency and reduce environmental pollution [27,28].

This study faced limitations due to time constraints, economic costs, and other factors affecting variable control and result interpretation. Future research can address these limitations by increasing sample sizes, improving methodologies, and collecting more comprehensive data.

## Figures and Tables

**Table 1:** XRF chemical compositions analysis of copper oxide ore (wt%).

SiO_2_	CaO	MgO	Al_2_O_3_	K_2_O	Fe_2_O_3_	CuO
56.1202	18.3875	11.6775	8.5244	1.6679	1.3900	1.2818
TiO_2_	P_2_O_5_	Co_2_O_3_	MnO	SO_3_	ZrO_2_	Na_2_O
0.5054	0.1725	0.1199	0.1054	0.0308	0.0141	0.0023

**Table 2:** Stability constants of related complex ions (25 °C).

Type	Stability Constant lgβ
Cu(NH_3_)^2+^	4.24
Cu(NH_3_)_2_^2+^	7.83
Cu(NH_3_)_3_^2+^	10.80
Cu(NH_3_)_4_^2+^	13.00
Cu(NH_3_)_5_^2+^	12.43
Cu(OH)^+^	6.30
Cu(OH)_2_(aq)	12.80
Cu(OH)_3_^−^	14.50
Cu(OH)_4_^2−^	15.60
Cu_2_(OH)_2_^2+^	17.28
Cu(NH_3_)_3_(OH)^+^	14.90
Cu(NH_3_)_2_(OH)_2_(aq)	15.70
Cu(NH_3_)(OH)_3_^−^	16.30
HCO_3_^−^	9.56
H_2_CO_3_	15.98
NH_4_^+^	9.80

## Data Availability

The original contributions presented in the study are included in the article, further inquiries can be directed to the corresponding authors.
